# Principal Factors Contributing to Cognitive Impairment in Homebound and Bedridden Frail Elderly Individuals from Low‐Resource Communities in Brazil

**DOI:** 10.1002/alz.090499

**Published:** 2025-01-09

**Authors:** ANA PAULA BERNARDES REAL, FLAVIA LANNA DE MORAES, EDGAR NUNES DE MORAES, CARLA JORGE MACHADO

**Affiliations:** ^1^ Jenny de Andrade Faria Institute – Reference Center for the Elderly, Federal University of Minas Gerais (UFMG), Belo Horizonte, Brazil, Belo Horizonte, MINAS GERAIS Brazil; ^2^ Jenny de Andrade Faria Institute – Reference Center for the Elderly, Federal University of Minas Gerais (UFMG), Belo Horizonte, Brazil, BELO HORIZONTE, Minas Gerais Brazil; ^3^ Jenny de Andrade Faria Institute – Reference Center for the Elderly, Federal University of Minas Gerais (UFMG), Belo Horizonte, Brazil, Belo Horizonte, Minas Gerais Brazil; ^4^ Faculty of Medicine, Federal University of Minas Gerais (UFMG), Belo Horizonte, Brazil, Belo Horizonte, Minas Gerais Brazil

## Abstract

**Background:**

Cognitive impairment (CI) is a major geriatric syndrome, leading to the loss of an individual’s decision‐making autonomy and representing a significant caregiving burden for families. It is commonly encountered in clinical practice and can be caused by dementia (major neurocognitive disorder), depression, delirium, or mental illness.

**Methods:**

To assess the prevalence and primary causes of cognitive impairment (CI), as well as its correlation with other clinical conditions among bed‐bound or home‐bound elderly individuals from low‐resource communities in Brazil, we carried out a cross‐sectional study. This study involved patients from a public health system’s home care service, who received care from specialized geriatric‐gerontological teams in the low‐income communities of Belo Horizonte/MG, from 2011 to 2022. Statistical analyses were performed using Stata/SE for Macintosh 12.0.

**Results:**

The study evaluated 1,391 elderly individuals, of which 67.8% were female, with an average age of 81.0 years (range 60 – 105). The prevalence of CI was 73.5%. The following probable etiologies were observed: dementia (82%), depression (40%), delirium (8.3%), and “mental illness” (15%), which were not mutually exclusive. The likelihood of CI among patients with delirium and mental illness was, respectively, 4.9 times (OR = 4.9; 95% CI 1.9‐12.8; p<0.001) and 7.6 times (OR = 7.6; 95% CI 4.1‐14.1; p<0.001) the likelihood of CI among patients without these conditions, indicating a significant and positive association. Age 80 years or older (OR = 1.6; 95% CI 1.2‐2.1; p = 0.003) and the presence of a high degree of frailty (OR = 2.2; 95% CI 2‐2.4) were also positively associated with CI. Polypharmacy was negatively associated with cognitive impairment (OR = 0.61; 95% CI 0.45; 0.82; p = 0.001).

**Conclusions:**

The prevalence of CI was quite high in the population evaluated, present in nearly three‐quarters of the elderly. Dementia was the primary cause, followed by depression, mental illness and delirium. The association between dementia and depression was significant, complicating differential diagnosis while delirium constitutes the least frequent cause of CI in community‐dwelling elderly individuals.

